# Trimodal prehabilitation and psychological outcomes in colorectal cancer surgery: preliminary findings from a single-center randomized trial

**DOI:** 10.3389/fsurg.2026.1821350

**Published:** 2026-07-10

**Authors:** Elisa Trevisol, Nicolò Fabbri, Antonio Pesce, Rosario Lordi, Maria Giulia Nanni, Rosangela Caruso, Luigi Grassi, Carlo Vittorio Feo

**Affiliations:** 1 Universita Degli Studi di Ferrara, Ferrara, Italy; 2 Department of General Surgery, Local Health Agency of Ferrara, Azienda Unità Sanitaria Locale di Ferrara, Ferrara, Italy; 3 Department of General Surgery, Local Health Agency of Ferrara, Ferrara, Italy; 4 Division of Exercise and Sports Medicine, Department of Sports Medicine, Azienda USL di Ferrara, University of Ferrara, Ferrara, Italy; 5 Department of Biomedical and Specialty Surgical Sciences, Institute of Psychiatry, University of Ferrara, Ferrara, Italy

**Keywords:** anxiety, colorectal cancer, enhanced recovery after surgery (ERAS), multimodal prehabilitation, psychological well-being, quality of life

## Abstract

**Introduction:**

Surgery for colorectal cancer (CRC) entails significant physical and psychological stress. This clinical trial evaluated whether a trimodal prehabilitation program combining exercise, nutritional support, and psychological counseling could reduce anxiety and depression while improving health-related quality of life (HRQoL) within an Enhanced Recovery After Surgery (ERAS) pathway.

**Methods:**

In this single-blind randomized controlled trial, an interim analysis was conducted on 45 patients undergoing elective CRC surgery, assigned to trimodal prehabilitation (*n* = 20) or standard ERAS care (*n* = 25). The primary outcome was the longitudinal change in perioperative anxiety severity from baseline (T-4 weeks) to long-term follow-up (T + 52 weeks post-surgery) assessed via the GAD-7 scale. Secondary outcomes included depressive symptoms (PHQ-9) across five time points (T-4 to T + 52 weeks), HRQoL (SF-36, EORTC QLQ-C30/CR29), and exploratory correlations between baseline physical performance (6MWT and CPET) and longitudinal psychological trajectories.

**Results:**

GAD-7 scores decreased from 6.2 ± 5.1 to 2.2 ± 3.1 in the intervention group and from 4.7 ± 4.8 to 2.9 ± 2.8 in controls (between-group *p* < 0.001). Perceived health improvement (SF-36 “Health Change”) at 52 weeks was 76.6 ± 26.6 in the intervention group vs. 74.8 ± 29.2 in controls (*p* < 0.00001). No significant between-group differences were observed in PHQ-9 scores over time (*p* = 0.437). Significant improvements were observed in emotional functioning and cancer-related fatigue domains of the EORTC QLQ-C30 (*p* < 0.05). Correlations between physical performance and psychological outcomes were not statistically significant.

**Conclusions:**

Trimodal prehabilitation within an ERAS pathway reduces anxiety and improves perceived health status in patients undergoing CRC surgery. Larger multicenter studies are warranted to confirm its effects on depression and long-term outcomes.

**Clinical trial registration:**

https://clinicaltrials.gov/study/NCT06443203, identifier NCT06443203.

## Introduction

**Figure 1:**
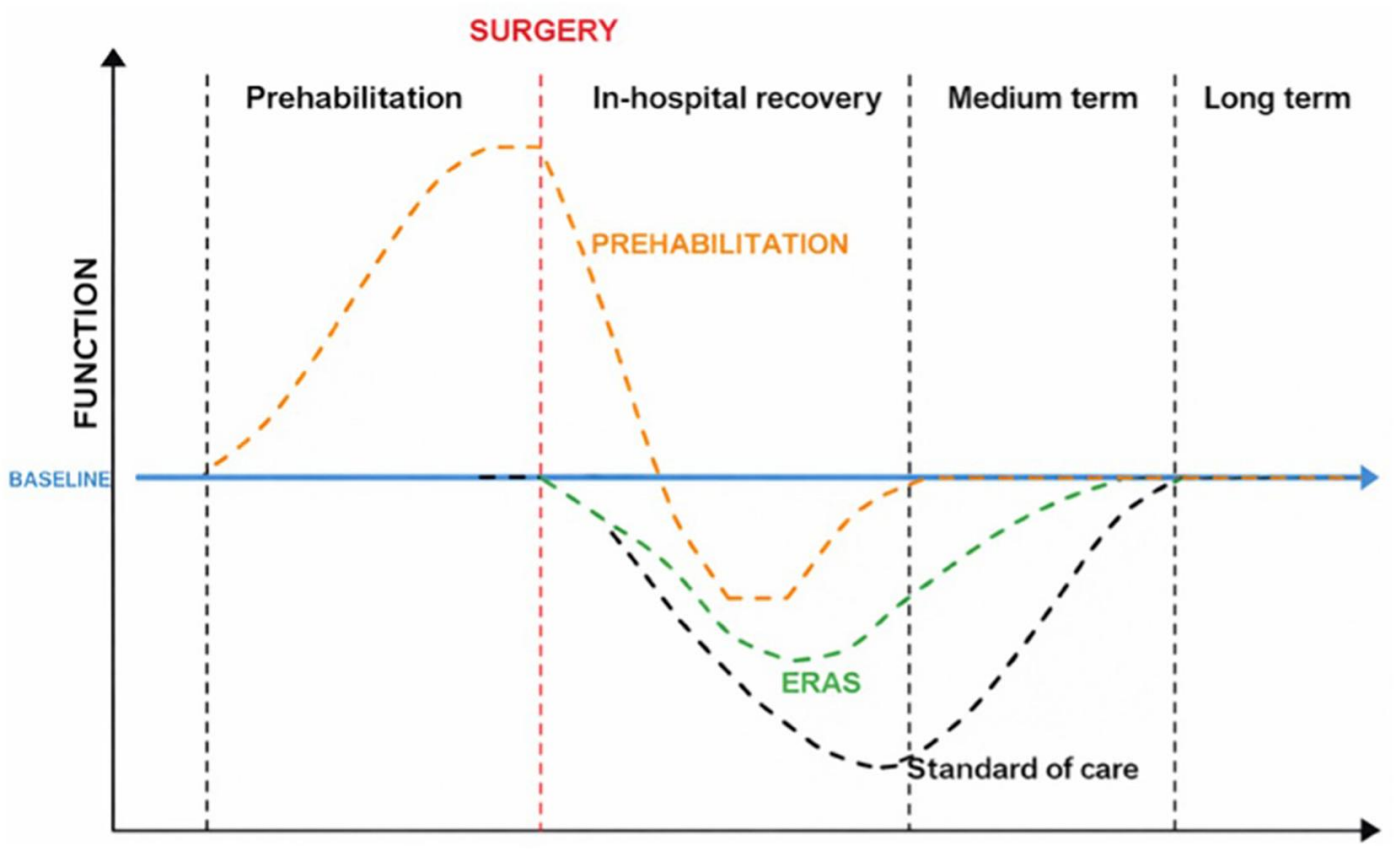
Comparison between the traditional approach, the ERAS protocol, and prehabilitation.

Colorectal cancer (CRC) is one of the most commonly diagnosed malignancies worldwide and a leading cause of cancer-related mortality (1). Despite improvements in screening and early detection, surgical resection remains the cornerstone of curative therapy (2), yet it is associated with substantial perioperative morbidity. Postoperative complications, including pain, fatigue, impaired bowel function, and delayed recovery, affect up to 50% of patients, with implications for overall outcomes and health-related quality of life (HRQoL) (3, 4). Even in uncomplicated courses, major abdominal surgery induces a 20%–40% decline in physical and psychological functional capacity, often persisting for weeks (5). Pre-existing factors such as malnutrition, smoking, psychological distress, and reduced baseline functional capacity further amplify postoperative vulnerability, particularly in elderly and frail patients. Enhanced Recovery After Surgery (ERAS) protocols have significantly improved perioperative outcomes by attenuating surgical stress and standardizing care (6), yet they may not fully address patient-related vulnerabilities, especially in the postoperative period when fatigue, anxiety, and depressive symptoms limit engagement in rehabilitative strategies. Prehabilitation, therefore, represents a paradigm shift from reactive postoperative recovery to proactive perioperative optimization. Implemented between diagnosis and surgery, multimodal prehabilitation aims to enhance functional and psychological reserve, thereby improving tolerance to surgical stress and facilitating recovery (7, 8). The conceptual model (Figure 1) illustrates how prehabilitation may elevate preoperative functional capacity, attenuate postoperative decline, and accelerate recovery, occasionally exceeding baseline function (9). While the physical benefits of preoperative exercise are well documented (7, 10, 11), its impact on postoperative complications and hospital stay remains controversial, with heterogeneous findings across studies (3, 12–15). Moreover, most prehabilitation research focuses on physical outcomes, often underestimating psychological distress such as anxiety and depression, which affects up to 40% of patients awaiting cancer surgery (14, 16). Trimodal prehabilitation programs, combining physical exercise, nutritional support, and psychological interventions, may therefore offer broader benefits (10, 11). By addressing psychological well-being, these programs aim to improve coping strategies and self-efficacy, potentially enhancing recovery. The present study contributes to the growing evidence supporting perioperative prehabilitation in colorectal cancer patients by investigating whether adding a structured psychological module to a trimodal program reduces perioperative anxiety and depression and improves long-term HRQoL in patients undergoing elective surgery (17).

## Materials and methods

A single-center, single-blind, randomized controlled trial was conducted at the Department of Surgery of the Azienda USL and the Azienda Ospedaliero-Universitaria of Ferrara, in northeastern Italy. The study followed the protocol “*Multimodal prehabilitation in colorectal cancer patient to iimprove functional capacity and reduce postoperative complications”*, funded by the Italian Ministry of Health (Project RF-2018-12367272, Emilia-Romagna Region). Written informed consent was obtained from all eligible patients prior to inclusion. The study adhered to the CONSORT guidelines (18) and was approved by the local Ethical Committee (Comitato Etico Area Vasta Emilia Centro – CE-AVEC; protocol ID: 849/2019/Sper/AUSLFe). All procedures were performed in accordance with the Declaration of Helsinki. The trial is registered at ClinicalTrials.gov (NCT06443203).

The present analysis was conducted as an exploratory interim analysis. Longitudinal changes in psychological and quality-of-life outcomes were assessed separately within the two groups using repeated-measures tests: the Friedman test for non-normally distributed variables and repeated-measures ANOVA when appropriate. Comparisons between time points within the same group were performed using the paired Student's t-test or the Wilcoxon signed-rank test. Comparisons between the intervention and control groups at individual time points were performed using the unpaired Student's t-test or the Mann–Whitney U test, according to data distribution. Categorical variables were compared using the chi-square test. Normality was assessed using the D’Agostino-Pearson test. Linear mixed models and formal group × time interaction analyses were not applied. Therefore, between-group comparisons at individual time points were interpreted as exploratory. Patients were eligible if they were older than 18 years, scheduled for elective colorectal cancer surgery, and managed according to the Enhanced Recovery After Surgery (ERAS) protocol. Exclusion criteria included included known metastatic disease, any state of immobilization, orthopedic or chronic conditions contraindicating physical activity, cognitive disabilities, chronic renal failure stage > 2, an ASA score of IV or higher, illiteracy, and planned rectal resection.

**Figure 2:**
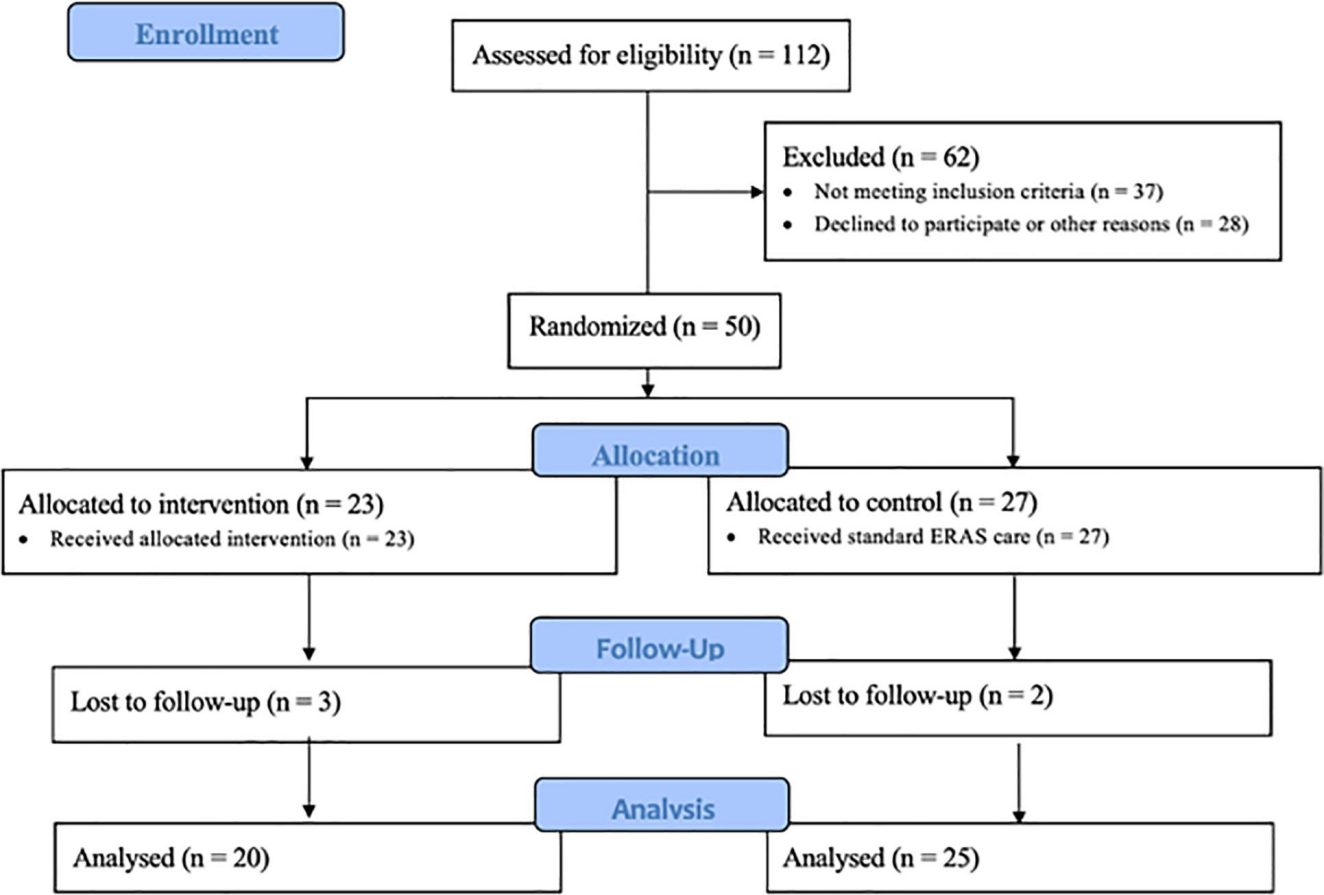
Consort flow diagram.

Initially, 50 patients were randomized. However, 5 patients were excluded from the final analysis due to incomplete 52-week follow-up. The final analysis was therefore conducted on 45 patients, including 20 in the intervention group and 25 in the control group, as detailed in the CONSORT flow diagram (Figure 2). Patients allocated to the intervention group underwent a 4-week trimodal prehabilitation program delivered by a multidisciplinary team, while the control group received standard perioperative care according to ERAS protocol (19). The intervention was structured and supervised by qualified healthcare professionals. The initial functional assessment, including cardiopulmonary exercise testing (CPET) and the Six-Minute Walk Test (6MWT), was performed and interpreted by a specialist in Physical Medicine and Rehabilitation. Exercise sessions were supervised by a physiotherapist or clinical exercise specialist, the nutritional plan was developed by a registered dietitian, and psychological support was provided by a licensed clinical psychologist.

The objective of this study was to evaluate whether a 4-week trimodal prehabilitation programme, including exercise training, nutritional optimization, and structured psychological support, compared with standard ERAS perioperative care, improves perioperative anxiety, depressive symptoms, and health-related quality of life in patients undergoing elective colorectal cancer surgery.

The primary outcome was the between-group difference evaluation of longitudinal changes in perioperative anxiety severity from baseline (T−4 weeks) to long-term follow-up (T + 52 weeks post-surgery), measured using the validated 7-item Generalized Anxiety Disorder scale (GAD-7). Secondary outcomes included the assessment of longitudinal trajectories of depressive symptoms using the 9-item Patient Health Questionnaire (PHQ-9), as well as changes in multidimensional health-related quality of life (HRQoL), evaluated through the Short Form-36 (SF-36), the EORTC QLQ-C30 generic cancer module, and the colorectal cancer-specific EORTC QLQ-CR29 module. Additionally, we examined the association between physical performance, assessed by the Six-Minute Walk Test (6MWT) and Cardiopulmonary Exercise Testing (CPET), and psychological status. This choice was motivated by our previous evidence showing that prehabilitation improves physical performance in patients with colorectal cancer (Pesce et al., 2024) (20), suggesting that improvements in physical function may be accompanied by enhanced psychological well-being (Table 1).

**Table 1:** Objective type.

Objective Type	Brief Description	Instruments Used
Primary	Evaluation of longitudinal changes in perioperative anxiety severity	GAD-7
Secondary	Assessment of longitudinal trajectories of depressive symptoms	PHQ-9
	Evaluation of multidimensional health-related quality of life (HRQoL)	SF-36; EORTC QLQ-C30; EORTC QLQ-CR29
	Correlation between psychological improvement and physical performance	GAD-7; PHQ-9; 6MWT; CPET

### Elements of trimodal prehabilitation

#### Exercise program

Patients underwent an initial functional assessment including cardiopulmonary exercise testing (CPET), the Six-Minute Walk Test (6MWT), and strength evaluations. Based on these results, a sports medicine physician defined and supervised an individualized 4-week exercise program consisting of three supervised sessions per week. Exercise intensity was prescribed according to CPET-derived parameters, ensuring full personalization. The program combined aerobic interval training and resistance exercises, complemented by home-based aerobic activity. Patients were instructed to perform additional unsupervised aerobic exercise (walking or cycling) at approximately 50% of their heart rate reserve, progressively increasing as tolerated toward a target of 60 min, four times per week. To ensure adherence, patients wore an accelerometer throughout the 4-week intervention period. CPET, considered the gold standard for the non-invasive assessment of cardiorespiratory fitness, was performed using a maximal incremental treadmill protocol (starting at 2.4 km/h and 1.5% incline, with progressive increases of 0.16 km/h and 0.5% incline every 30 s) (21). Patients were instructed to avoid food or beverages (except water) for at least 2 h before testing and to refrain from physical exercise for 48 h prior to assessment. Pharmacological therapy was continued as usual. The primary outcome of CPET was VO₂max, a biomarker of functional capacity and oxygen delivery efficiency. The 6MWT was used as a complementary measure of functional exercise capacity. Patients walked along a 30-meter corridor for 6 min, and the total distance covered (6MWD) was recorded (22, 23).

#### Nutritional supplements

Nutritional status was evaluated using a food diary, the Patient-Generated Subjective Global Assessment (PG-SGA), and anthropometric and body composition measurements. At baseline (time zero), a dietitian examined the patient's caloric balance and dietary intake according to the ESPEN Clinical Guidelines on Clinical Nutrition and Surgery (24).

A personalized nutritional plan was prescribed with the aim of achieving an anabolic state in all patients and improving lean body mass, particularly in cachectic individuals. Protein intake was determined at1.5 - 1.8 g/kg/day and adapted to the patient's habitual food intake and nutritional status. In cases of weight loss or cachexia, and in the absence of medical contraindications, protein intake was increased to at least 1.5 g/kg/day. Nutritional supplementation included whey protein, casein, omega-3 fatty acids, and vitamin D.

#### Psychological coping

All patients underwent psychometric screening using the Generalized Anxiety Disorder-7 (GAD-7) to assess anxiety symptoms and the Patient Health Questionnaire-9 (PHQ-9) to evaluate depressive symptoms. Patients scoring ≥10 on the GAD-7 or ≥15 on the PHQ-9 were eligible for a structured psychological support program. This intervention included: 1) An initial 90-minute individual session, with 60 min dedicated to emotional assessment and psychological exploration and 30 min to relaxation techniques (diaphragmatic breathing, progressive muscle relaxation, guided imagery), supported by home-based practice materials; 2) Weekly follow-up sessions for monitoring and reinforcement of coping strategies; 3) Psychoeducational interventions aimed at enhancing understanding of the surgical pathway and patient self-efficacy; 4) Involvement of caregivers, when appropriate, to promote relational support and adherence to recommendations.

To comprehensively evaluate psychological status and health-related quality of life (HRQoL), patients completed five internationally validated self-report questionnaires at multiple time points:

T-4 weeks (baseline, before the start of the prehabilitation program)
T-1 week (one week before surgery, end of the program
T + 4 weeks (4 weeks after surgery)
T + 8 weeks (8 weeks after surgery)
T + 52 weeks (12 months after surgery)

The questionnaires were administered either in paper format or by telephone; if difficulties arose, support was provided to ensure integrity of responses.The following section provides a detailed description of the psychometric instruments adopted in the study (Table 2):

**Table 2:** Overview of the questionnaires.

Questionnaires	Number of Items & Score Range	Key Categories & Cut-offs
GAD-7	7 items; Total score 0–21	0–4 = no or minimal; 5–9 = mild; 10–14 = moderate; ≥ 15 = severe
PHQ-9	9 items; Total score 0–27	0–4 = no or minimal; 5–9 = mild; 10–14 = moderate; 15–19 = moderately severe; ≥ 20 = severe
SF-36	36 items; Scores 0–100	Two summary scores: General Health and Health Change (higher scores = better health status);
EORTC QLQ-C30	Cancer-specific module; 1 global scale, 5 functional scales, 3 symptom scales, 6 single items; Scores 0–100	Global and functional scales (higher scores = better QoL); symptom scales and items (higher scores = worse QoL)
EORTC QLQ-C29	Colorectal cancer-specific module; 29 items; Scores 0–100	Higher scores = worse QoL

##### GAD-7 (generalized anxiety disorder-7)

A 7 - item instrument developed by Spitzer et al. To assess the frequency over the past 14 days of symptoms such as nervousness, difficulty relaxing, and persistent worry. Each item is rated on a 4 - point Likert scale from 0 (“not at all”) to 3 (“nearly every day”), yielding a total score from 0 to 21; higher scores indicate greater anxiety severity. A cut-off of ≥ 10 is frequently used to identify clinically significant generalized anxiety disorder (25).

##### PHQ-9 (patient health questionnaire-9)

A 9 - item self-report questionnaire based on DSM-IV criteria for depression, exploring emotional, cognitive, somatic and motivational symptoms (depressed mood, anhedonia, sleep disturbance, fatigue, suicidal thoughts). Each item is scored 0 to 3 (0 = “not at all”, 3 = “nearly every day”), giving a total score from 0 to 27. Commonly used cut-offs are: 0 - 4 (Minimal or no depression), 5–9 (mild depression), 10–14 (moderate depression), 15–19 (moderately severe depression), ≥ 20 (severe depression) (26).

##### HRQoL (health-related quality of life) questionnaires

Three validated instruments were employed:

- **SF-36 (Short Form 36):** Comprises 36 items assessing eight dimensions (physical functioning, role limitation due to physical health, role limitation due to emotional problems, energy/fatigue, emotional well-being, social functioning and pain). Two summary scores are derived: “General Health” and “Health Change” (27).
- **EORTC QLQ-C30:** A cancer-generic module which includes one global quality of life scale, 5 functional scales (physical, role, emotional, cognitive, social), three symptom scales (fatigue, pain, nausea/vomiting) and 6 single items (dyspnoea, insomnia, appetite loss, constipation, diarrhoea, financial difficulties) (28).
- **EORTC QLQ-CR29:** A colorectal-cancer-specific module with 29 items: 4 multi-item scales (anxiety, body image, male/female sexual function) and 11 single-item questions covering gastrointestinal, urological and relational/emotional symptoms. In both EORTC modules the scores are transformed to a 0-100 scale (for functional scales: higher = better quality of life; for symptom scales: higher = greater symptom burden) (29).

### Statistical analysis

The questionnaires used in the study were evaluated according to the scoring criteria specified for each instrument. For the PHQ-9 and GAD-7 questionnaires, which measure depressive symptoms and anxiety symptoms, respectively the raw scores were used (range 0–27 for PHQ-9; 0–21 for GAD-7). Clinical thresholds were interpreted as follows:

- **GAD-7:** 0–4 = minimal anxiety; 5–9 = mild; 10–14 = moderate; ≥ 15 = severe.
- **PHQ-9:** 0–4 = no or minimal depression; 5–9 = mild; 10–14 = moderate; 15–19 = moderately severe; ≥ 20 = severe.

For the SF-36, the scores of the eight principal scales were transformed onto a 0–100 scale, where higher values indicate better quality of life; in the present study, the “General Health” and “Health Change” summary scores were used, calculated via the online Orthotoolkit calculator (30). For the EORTC QLQ-C30 and EORTC QLQ-CR29 modules, functional and symptom scales were calculated in accordance with the EORTC scoring manual, with a linear transformation to a 0–100 scale: for functional scales, higher values indicate better quality of life; for symptom scales, higher values indicate greater symptom burden (28, 31). The statistical analysis of the study examined the effect of a psychological prehabilitation program integrated into the ERAS protocol on a population of patients undergoing surgery for colorectal cancer, with questionnaire administrations at five perioperative time points (T−4, T−1, T + 4, T + 8and T + 52). The chi-square test was used for group comparisons of categorical variables (e.g., sex, presence of comorbidities). Normality of quantitative variables was tested using the D’Agostino- Pearson test. Within-group comparisons between different time points (e.g., T−4 vs. T−1) were performed using the paired Student's t-test for normally distributed data or the Wilcoxon test otherwise. Between-group comparisons at the same time point or at different time points (e.g., T−4 intervention group vs. T−4 control group) used the unpaired Student's t-test for normally distributed data or the Mann–Whitney test for non-normal data. For analysis of repeated measures over time, repeated-measures ANOVA (for normal distribution) or the Friedman test (for non-normal distribution) was employed. All statistical models were adjusted for relevant sociodemographic and clinical baseline variables considered invariant over time, including age, sex, body mass index (BMI), ASA score and presence of comorbidities. Differences were considered statistically significant if *p* < 0.05. The analysis was performed using MedCalc version 20.111 (Ostend, Belgium, 2022).

## Results

In the current study, an interim exploratory analysis of 45 patients who completed 12-month follow up (T + 52 weeks) undergoing colorectal surgery was performed, with 20 assigned to interventional arm and 25 to control arm, as shown in CONSORT flow diagram (Figure 2). No formal sample size calculation was performed specifically for the psychological and HRQoL endpoints reported in the present analysis. Therefore, the statistical analyses should be interpreted as exploratory and hypothesis-generating. Demographic and clinical data are reported in Table 3. Baseline characteristics were comparable in both groups. The intra-operative characteristics of the two groups were overall comparable (Table 4), with a higher use of peripheral nerve blocks in the intervention group (65% vs. 48%). Postoperatively, ICU admission was more frequent in the intervention group (15% vs. 4%). Recovery of oral intake occurred earlier in the control group, both for liquids (1.4 ± 0.7 vs. 2.3 ± 2.8 days) and solid food (2.3 ± 0.9 vs. 3.1 ± 3.0 days). Vomiting after 24 h was more common in the intervention group (25% vs. 4%). Length of hospital stay was similar between groups. Despite comparable complication rates by Clavien-Dindo classification, the overall postoperative burden was lower in the intervention group, as demonstrated by a reduced Comprehensive Complication Index (5.61 ± 11.82 vs. 11.12 ± 21.8) (Table 5). Patient-reported outcomes were evaluated longitudinally at T−4, T−1, T + 4, T + 8 and T + 52 weeks (Table 6). Analyses were based on between-group comparisons over time (group  ×  time effects where applicable).

**Table 3:** Sample characteristics.

Sample characteristics	All	Intervention group	Control group	*p* value
Number of patients	45	20	25	
*Gender [N (%)]*				0,944
Male	29 (64,4%)	13 (65%)	16 (64%)	
Female	16 (35,6%)	7 (35%)	9 (36%)	
Age (years) ± SD	67,8 ± 9,0	65,5 ± 8,0	69,4 ± 9,5	0,172
BMI (Kg/m2) ± SD	28,5 ± 4,8	28,8 ± 3,7	28,2 ± 5,5	0,704
Barthel Index (score) ± SD	99,9 ± 0,8	100 ± 0	100 ± 1	
CIRS ± SD	5,6 ± 1,8	6 ± 1,8	6 ± 1,8	
*ASA score [N (%)]*				0,926
1	0	0	0	
2	16 (36%)	7 (35%)	9 (36%)	
3	26 (58%)	11 (55%)	15 (60%)	
4	0	0	0	
CCI ± SD	4,7 ± 1,2	4,4 ± 1,2	4,9 ± 1,2	0,305
Coronary disease [N (%)]	21 (47%)	10 (50%)	11 (44%)	0,688
Peripheral vascular disease [N (%)]	2 (4,4%)	1 (5%)	1 (4%)	0,871
Cerebrovascular disease [N (%)]	1 (2,2%)	0	1 (4%)	0,662
BPCO [N (%)]	1 (2,2%)	1 (5%)	0	0,423
Connective tissue disease [N (%)]	1 (2,2%)	0	1 (4%)	0,662
Peptic ulcer [N (%)]	2 (4,4%)	1 (5%)	1 (4%)	0,871
Liver disease [N (%)]	1 (2,2%)	1 (5%)	0	0,423
Diabetes mellitus [N (%)]	3 (6,6%)	1 (5%)	2 (8%)	0,688
Kidney failure [N (%)]	1 (2,2%)	0	1 (4%)	0,662
VO_2_ *peak* mL*kg*min	20,8 ± 6,9	23,1 ± 6,4	19,0 ± 7,0	0,563
6 MWD m	464 ± 112,0	496 ± 98	432 ± 114	0,076
*PG-SGA score [N (%)]*				
- 4 weeks				0.938
A [N (%)]	34 (75,6%)	15 (75%)	19 (76%)	
B [N (%)]	11 (24,4%)	5 (25%)	6 (24%)	
Preoperative haemoglobin (g/dL) ± SD	14,3 ± 0,4	12,8 ± 2,5	14 ± 2	0,673
Preoperative glucose (mg/dL) ± SD	100,6 ± 25,1	108,3 ± 15,2	94,9 ± 29,5	0,931

SD, standard deviation; N (%), number of patients (percentage); BMI, Body Mass Index; CIRS, Cumulative Illness Rating Scale; ASA, American Society of Anesthesiologists; CCI, Charlson Comorbidity Index; COPD, chron.

**Table 4:** Intraoperative characteristics of the sample.

Intraoperative characteristics	Intervention group	Control group	*p* value
Epidural catheter insertion [N (%)]	3 (15%)	5 (25%)	0,663
Peripheral nerve block [N (%)]	13 (65%)	12 (48%)	0,254
Total Intraoperative infusions (mL) ± DS	2352,9 ± 765,8	2470,5 ± 624,2	0,355
PONV prophylaxis [N (%)]	8 (40%)	13 (52%)	0,423
Blood loss (mL) ± DS	65,0 ± 68,9	61,4 ± 68,1	0,759
Operative time (min) ± DS	210,6 ± 31,9	232,7 ± 64,1	0,563
NGT in place at completion [N (%)]	11 (55%)	12 (48%)	0,641

SD, standard deviation; N (%), number of patients (percentage); PONV, postoperative nausea and vomiting; NGT, nasogastric tube.

**Table 5:** Postoperative characteristics of the sample.

Postoperative characteristics	Intervention group	Control group	*p* value
ICU admission [N (%)]	3 (15%)	1 (4%)	0,198
Day of Foley catheter removal ± DS	1,8 ± 0,83	1,9 ± 0,9	0,668
Day of oral liquid intake OS ± DS	2,3 ± 2,8	1,4 ± 0,7	0,284
Day of oral solid intake OS ± DS	3,1 ± 3,0	2,3 ± 0,9	0,721
Vomiting within 24 h [N (%)]	3 (15%)	3 (12%)	0,769
Day of bowel movement (stools) ± DS	3,0 ± 1,2	3,0 ± 1,2	0,289
Day of independent mobilization ± DS	3,0 ± 4,0	2,4 ± 0,9	0,847
Day of discharge ± DS	5,7 ± 6,4	5,9 ± 4,3	0,244
Clavien-Dindo Scale			
Grade II	3 (15%)	3 (12%)	0.769
Grade IIIa	0 (0%)	1 (4%)	0.899
Grade IIIb	1 (5%)	1 (4%)	0.872
Grade IVa	0 (0%)	0 (0%)	
Grade IVb	0 (0%)	0 (0%)	
Grade V	0 (0%)	1 (4%)	0.899
Comprehensive Complication Index (CCI)	5,61 ± 11,82	11,12 ± 21,8	0,332

SD, Standard Deviation; N (%), number of patients (percentage); ICU, Intensive Care Unit; PO, by mouth; CCI, Comprehensive Complication Index.

**Table 6:** Overview of the results of the GAD-7, PHQ-9, SF-36, EORTC QLQ-C30, and QLQ-CR29 questionnaire.

Questionnaires	Intervention group					Control group					*p* value
Perioperative time points	T-4	T-1	T + 4	T + 8	T + 52	T-4	T-1	T + 4	T + 8	T + 52	
GAD-7											
Anxety	6,2 ± 5,1	5,5 ± 4,2	3,8 ± 4,7	3,3 ± 3,8	2,2 ± 3,1	4,7 ± 4,8	4,3 ± 3,8	3,6 ± 2,6	2,8 ± 3,0	2,9 ± 2,8	**<0,001**
PHQ-9											
Depression	3,8 ± 2,6	3,6 ± 2,9	4,4 ± 3,7	3,9 ± 5,3	5,0 ± 6,2	3,6 ± 3,0	2,8 ± 2,5	11,5 ± 2,1	3,9 ± 3,9	3,2 ± 4,7	0,437
Functional impairment	1,5 ± 0,8	1,4 ± 0,5	1,5 ± 0,5	1,4 ± 0,5	1,3 ± 0,5	1,3 ± 0,5	1,2 ± 0,4	1,4 ± 0,6	1,4 ± 0,5	1,4 ± 0,6	0,188
SF - 36											
General health	58,9 ± 19,9	63,7 ± 19,0	68,7 ± 14,1	64,4 ± 24,7	64,3 ± 16,3	62,1 ± 16,7	61,5 ± 17,9	56,9 ± 22,9	57,4 ± 20,0	67,0 ± 18,6	0,155
Health changing	36,8 ± 17,4	37,5 ± 21,4	45,0 ± 25,4	55,6 ± 29,1	76,6 ± 26,6	34,8 ± 21,0	34,4 ± 23,5	45,2 ± 34,1	45,2 ± 32,2	74,8 ± 29,2	**<0,00001**
EORTC QLQ C-30											
Physical functioning	92,3 ± 10,7	91,8 ± 11,3	87,4 ± 13,1	92,6 ± 9,6	95,4 ± 8,3	82,7 ± 17,2	85,4 ± 14,8	77,7 ± 19,2	83,3 ± 17,1	88,8 ± 16,2	**<0,001**
Role Functioning	93,0 ± 14,0	96,3 ± 9,1	84,3 ± 16,0	92,6 ± 13,0	96,9 ± 9,0	89,5 ± 16,9	88,9 ± 15,2	74,6 ± 26,6	86,9 ± 15,8	88,4 ± 17,7	**<0,001**
Emotional functioning	79,9 ± 13,9	81,1 ± 17,3	82,9 ± 14,8	88,4 ± 14,3	94,8 ± 10,4	82,3 ± 18,0	85,8 ± 29,7	85,9 ± 14,9	86,7 ± 16,0	89,9 ± 13,3	**<0,001**
Cognitive functioning	91,1 ± 10,3	90,7 ± 13,0	87,3 ± 17,1	95,3 ± 7,8	96,9 ± 9,0	92,0 ± 11,1	90,9 ± 12,0	86,6 ± 12,9	86,2 ± 13,8	92,0 ± 12,1	0,202
Social functioning	97,3 ± 6,4	92,6 ± 17,4	85,4 ± 21,9	93,5 ± 14,1	95,8 ± 9,6	90,6 ± 15,7	93,8 ± 12,7	82,7 ± 20,4	92,7 ± 12,1	90,6 ± 15,7	0,633
Global health status	66,2 ± 24,9	76,9 ± 21,0	68,2 ± 24,4	72,1 ± 30,2	84,4 ± 22,2	71,6 ± 20,6	68,7 ± 20,0	68,0 ± 21,7	69,2 ± 21,2	78,8 ± 20,1	0,209
Cancer-Related Fatigue	11,6 ± 15,3	9,8 ± 13,5	19,4 ± 14,3	13,5 ± 11,7	8,9 ± 11,5	21,4 ± 17,9	17,5 ± 18,4	29,3 ± 20,1	18,7 ± 15,7	13,9 ± 16,0	**0,002**
EORTC QLQ CR-29											
Anxiety	43,7 ± 22,5	31,4 ± 21,4	33,1 ± 16,8	19,5 ± 16,8	12,5 ± 20,6	29,1 ± 24,8	30,5 ± 27,8	30,3 ± 24,5	21,7 ± 23,8	17,3 ± 19,7	**<0,001**
Weight	17,5 ± 23,2	9,2 ± 19,2	11,7 ± 20,2	5,8 ± 13,0	6,2 ± 13,3	22,2 ± 25,4	12,4 ± 19,1	13,0 ± 21,9	11,5 ± 19,0	2,9 ± 9,5	0,166
Body image	2,5 ± 4,7,0	2,4 ± 6,0	8,4 ± 12,6	3,2 ± 6,5	0,0 ± 0,0	10,1 ± 18,0	6,4 ± 11,7	8,6 ± 13,7	5,7 ± 9,3	4,8 ± 11,9	0,376
Sore skin	10,4 ± 20,1	3,9 ± 16,2	0,0 ± 0,0	0,0 ± 0,0	0,0 ± 0,0	3,0 ± 9,7	1,4 ± 6,9	1,6 ± 7,2	0,0 ± 0,0	0,0 ± 0,0	0,458

Bold values indicate statistically significant changes over time or between groups (*p* < 0.05).

**Figure 3:**
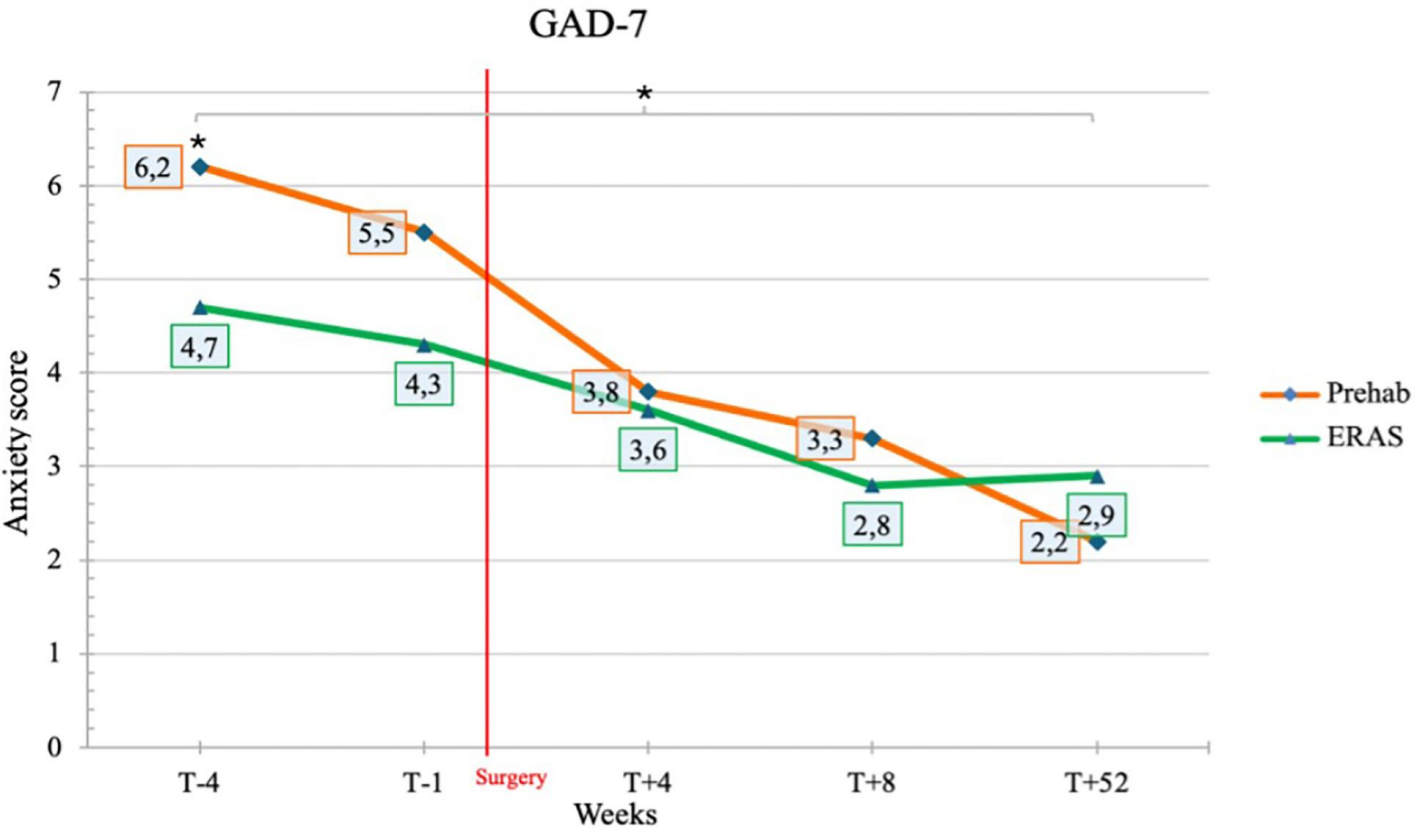
Trend of mean scores of the two groups in the anxiety scale of the GAD-7 across the five assessment time points. *p < 0,05.

**Figure 4:**
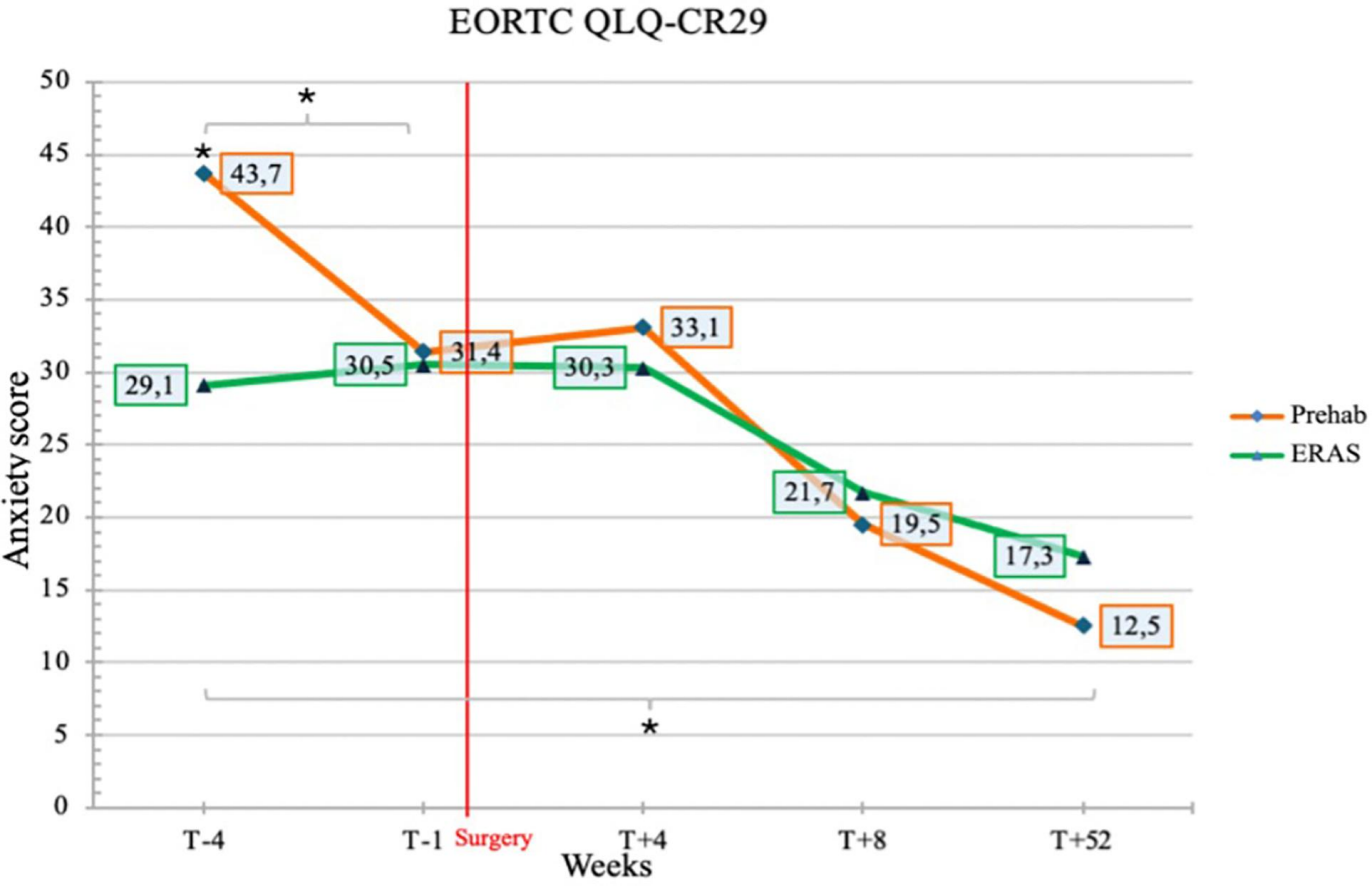
Trend of mean scores of the two groups in the anxiety scale of the EORTC QLQ-CR29 across the five assessment time points. *p < 0,05.

Anxiety: GAD-7 scores decreased over time in both groups, from 6.2 ± 5.1 at T−4 to 2.2 ± 3.1 at T + 52 in the intervention group, and from 4.7 ± 4.8 to 2.9 ± 2.8 in the control group (group-by-time comparison, *p* < 0.001) (Figure 3). A similar trend was observed for the EORTC QLQ-CR29 anxiety item, with reductions from 43.7 ± 22.5 to 12.5 ± 20.6 in the intervention group and from 29.1 ± 24.8 to 17.3 ± 19.7 in the control group (*p* < 0.001) (Figure 4).

Depression: in PHQ-9 no statistically significant between-group differences were observed in PHQ-9 scores over time (*p* = 0.437). Although transient fluctuations were observed at intermediate time points, these were not sustained across follow-up.

**Figure 5:**
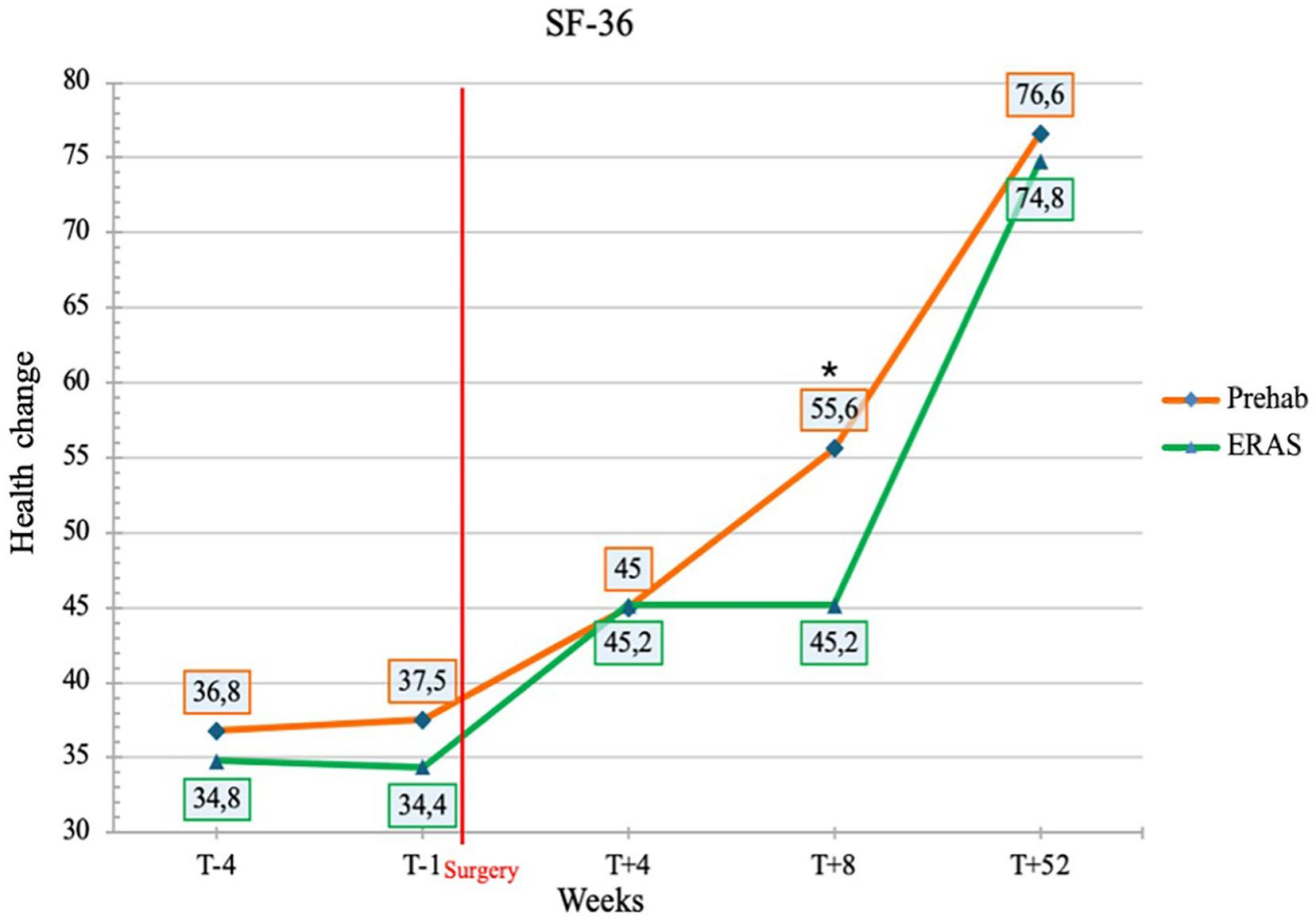
Trend of mean scores of the two groups in the health change scale of the SF-36 across the five assessment time points. *p < 0,05.

**Figure 6:**
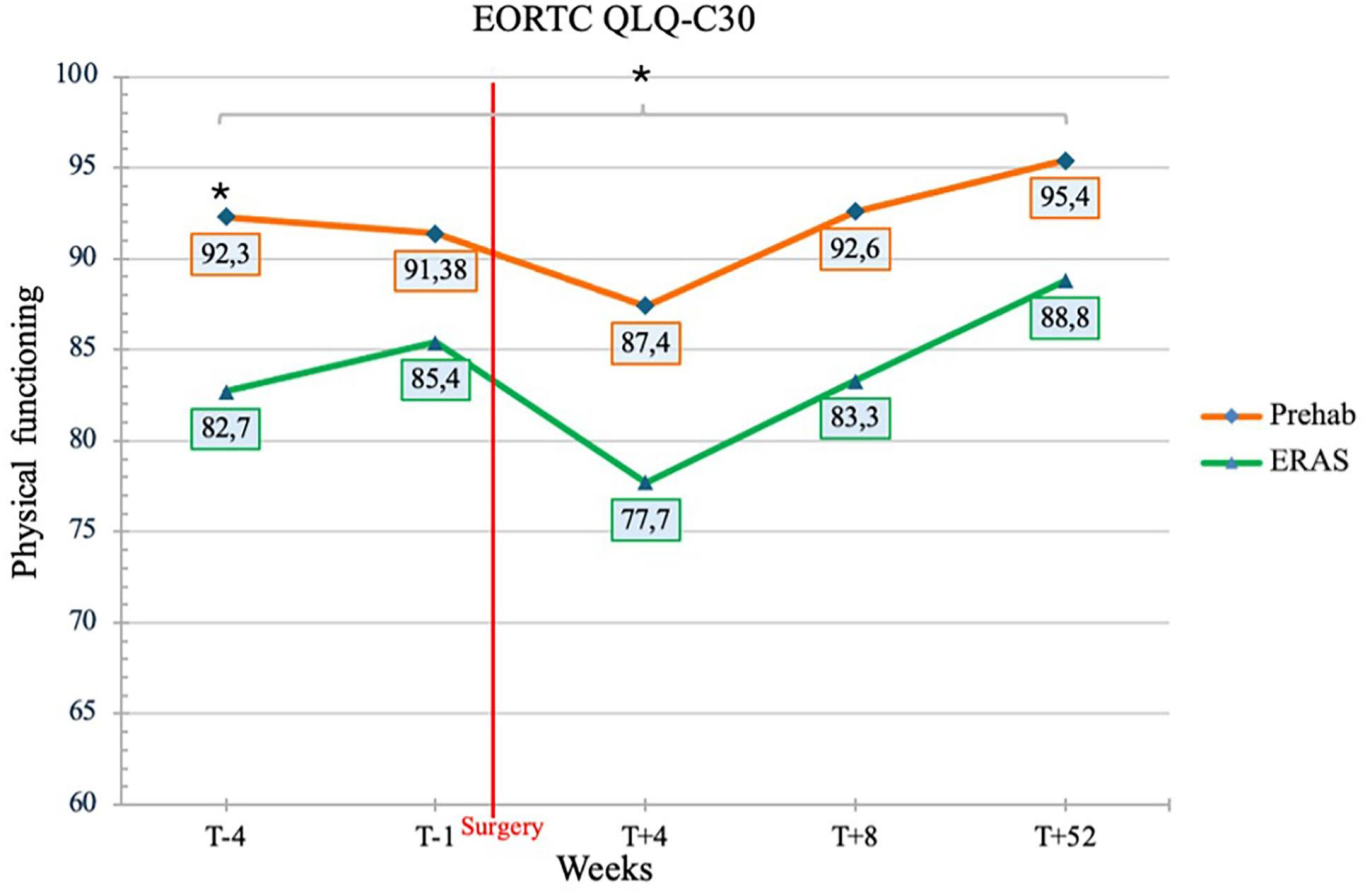
Trend of mean scores of the two groups in the physical functioning scale of the EORTC QLQ-C30 across the five assessment time points. *p < 0,05.

**Figure 7:**
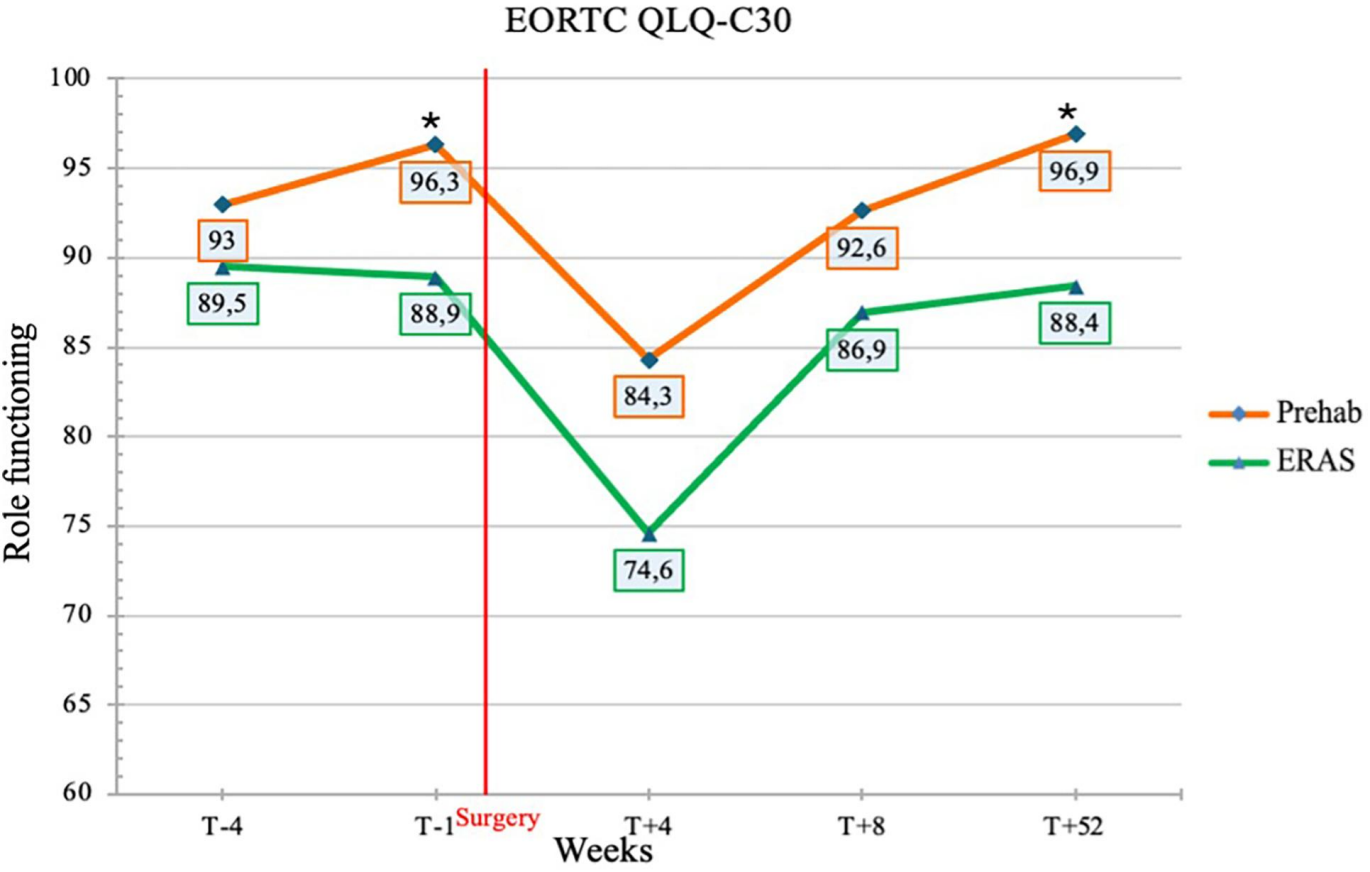
Trend of mean scores of the two groups in the role functioning scale of the EORTC QLQ-C30 across the five assessment time points. *P* < 0,05.

**Figure 8:**
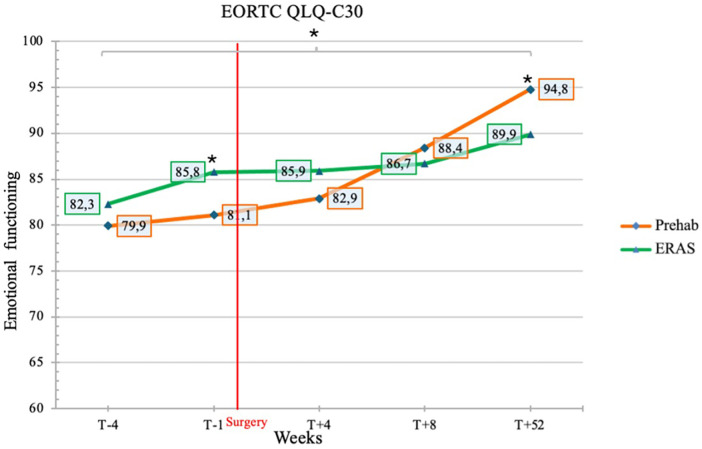
Trend of mean scores of the two groups in the emotional functioning scale of the EORTC QLQ-C30 across the five assessment time points. *P* < 0,05.

**Figure 9:**
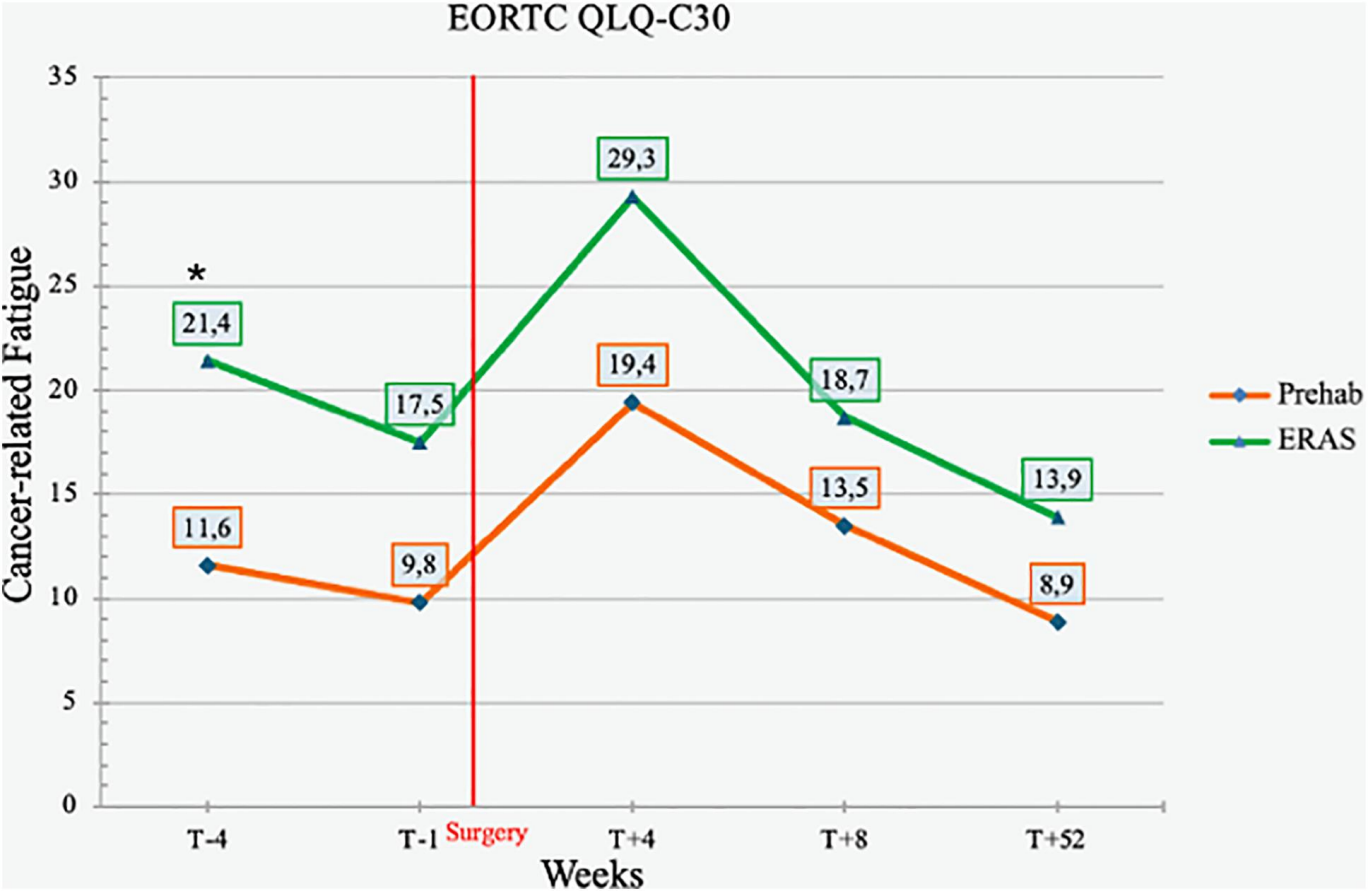
Trend of mean scores of the two groups in the CRF item of the EORTC QLQ-C30 across the five assessment time points. *P* < 0,05.

Quality of life: SF-36 “Health Change” improved in both groups over time, with no significant between-group difference (Figure 5; *p* < 0.00001 for within-group change; no significant interaction). “General Health” did not differ between groups (*p* = 0.155). In the EORTC QLQ-C30, physical functioning, role functioning, and emotional functioning showed group-by-time differences favouring the intervention group (*p* < 0.001) (Figures 6–8). Cancer-related fatigue also showed a reduction in the intervention group (*p* = 0.002) (Figure 9). No significant differences were observed for cognitive functioning, social functioning, or global health status. In the other items of EORTC QLQ-CR29 (body image, weight concern, perianal irritation and sexual interest domains) no significant between-group differences were observed.

**Figure 10:**
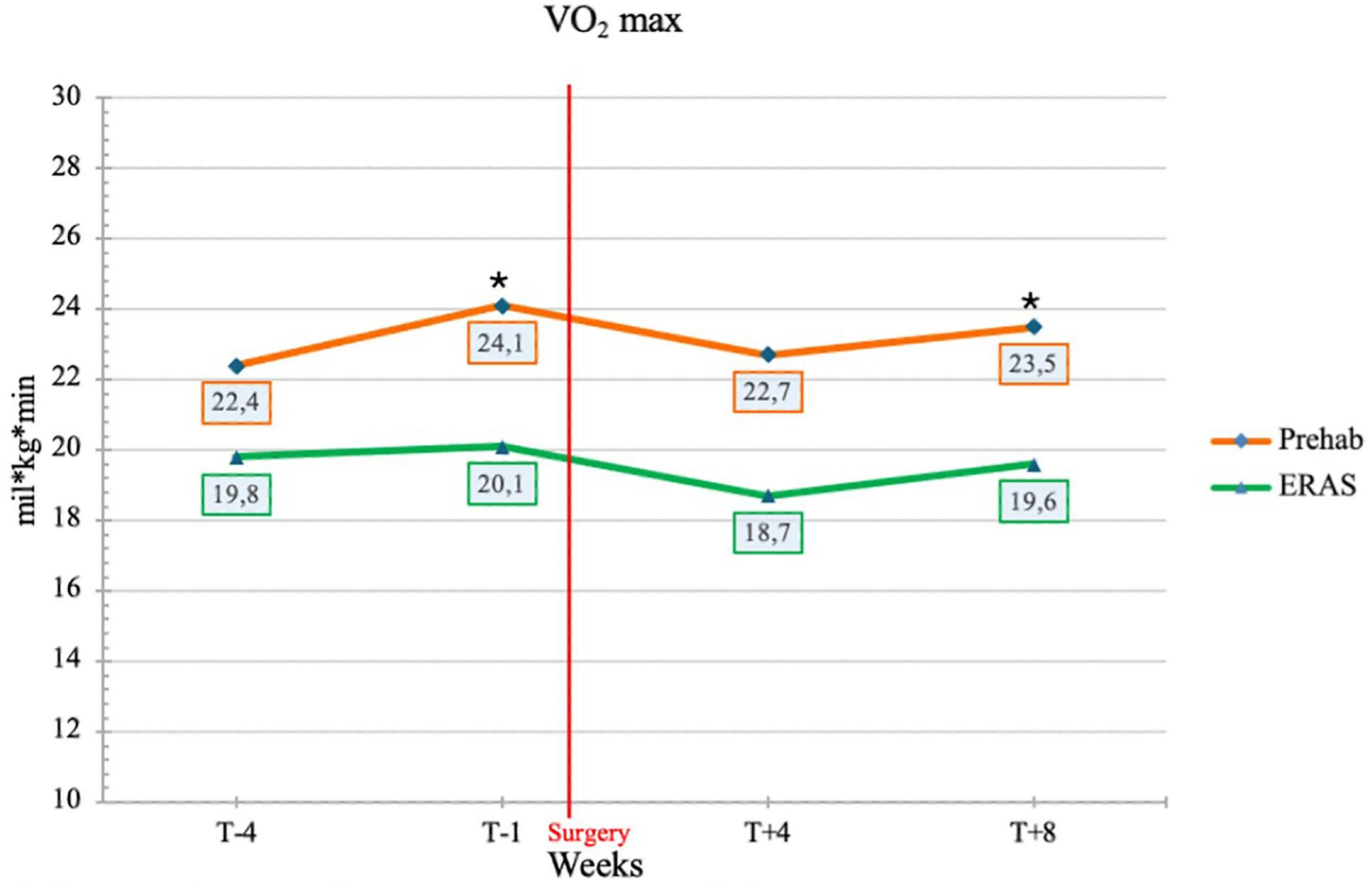
Trend of mean scores of the two groups in the CPET. *p < 0,05.

**Figure 11:**
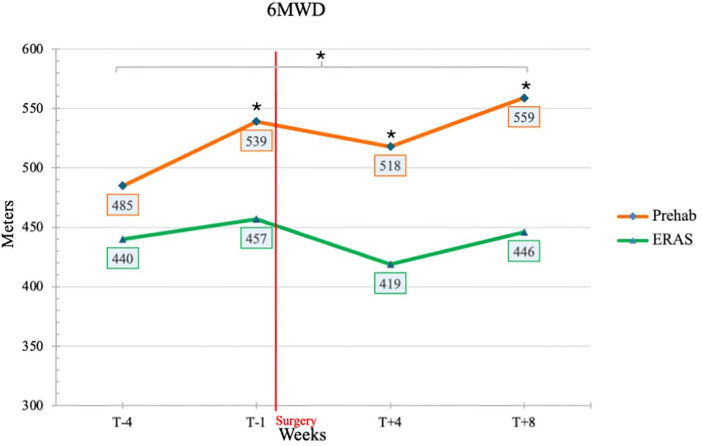
Trend of mean scores of the two groups in the 6MWT. *p < 0,05.

Functional outcomes: baseline values were comparable between groups. In CPET, VO₂ peak increased during the preoperative period in the intervention group (22.4 ± 3.2 to 24.1 ± 3.7 mL/kg/min), followed by a postoperative reduction and partial recovery. In the control group, VO₂ peak remained lower at all time points (19.8 ± 3.4 to 19.6 ± 2.6 mL/kg/min). In 6MWT Distance walked increased preoperatively in the intervention group (447.3 ± 44.2 to 492.7 ± 41.5 m), followed by a postoperative reduction and recovery. The control group showed lower values throughout follow-up (404.3 ± 47.8 to 412.4 ± 44.0 m). Between-group differences were statistically significant at T−1, T + 4, and T + 8 for both CPET and 6MWT (*p* < 0.05). (Figures 10, 11).

## Discussion

The present study suggests that integrating a trimodal prehabilitation program within an ERAS (Enhanced Recovery After Surgery) pathway may be associated with more favourable psychological trajectories and improved perceived recovery in patients undergoing colorectal cancer surgery. While ERAS protocols already represent a well-established standard of perioperative care, the addition of structured exercise, nutritional optimization, and psychological support appears to complement conventional perioperative management by addressing patient-level vulnerability factors that are often under-recognized in standard pathways.

In this exploratory randomized cohort, anxiety symptoms showed a progressive reduction over time in both groups, with a more pronounced decrease observed in patients receiving prehabilitation, particularly at long-term follow-up. This pattern was consistently observed across different anxiety-related measures. In contrast, depressive symptoms appeared more heterogeneous over time and did not show statistically significant between-group differences. These findings suggest that anxiety-related dimensions may be more sensitive to perioperative interventions targeting coping strategies and perceived control, whereas depressive symptoms may be influenced by a broader set of psychosocial and clinical factors not fully captured by the present intervention.

Health-related quality of life outcomes further supports this interpretation. Improvements in patient-reported “health change” were observed in both groups over time, with higher values at long-term follow-up in the intervention group, suggesting a more favourable perception of recovery trajectory. Similarly, selected functional and symptom domains of the EORTC QLQ-C30, including emotional functioning and cancer-related fatigue, demonstrated more favourable longitudinal patterns in the intervention group. However, other HRQoL domains, including cognitive and social functioning, did not differ between groups, highlighting a domain-specific rather than global effect of the intervention.

Importantly, improvements in physical performance measures, including VO₂ peak and 6-minute walk distance, were more pronounced in the intervention group during the preoperative period, consistent with previous evidence supporting the role of exercise-based prehabilitation in enhancing functional capacity. However, exploratory analyses did not demonstrate statistically significant correlations between physical performance and psychological outcomes. This finding suggests that the psychological benefits observed in the intervention group may not be solely mediated by improvements in physical fitness but rather reflect a broader biopsychosocial effect of multimodal prehabilitation.

These findings are consistent with previous literature supporting multimodal approaches. Frutos-Reoyo et al. (32), in a recent case–control study, reported improvements in functional capacity following prehabilitation, in line with the objective measures observed in our population (CPET and 6MWT). Although the primary focus of our study was on psychological outcomes, the parallel improvement in physical and emotional domains reinforces the concept of trimodal prehabilitation described by Scheede-Bergdahl et al. (4), where both physiological and behavioural components are considered essential in surgical oncology pathways.

An interesting aspect of our results is the different impact on anxiety and depressive symptoms. While anxiety showed a clear and progressive reduction, the effect on depression was less pronounced. This distinction has also been noted in previous studies. Dana F. et al. (33), for example, highlighted that improvements in quality of life may not be uniform across all domains. In our sample, patients undergoing prehabilitation did not exhibit the transient increase in depressive symptoms observed in the control group at T + 4, suggesting a potential buffering effect of the intervention, even in the absence of statistically significant differences in PHQ-9 scores. This observation adds nuance to the findings of Hijazi et al. (13) who, in their systematic review, confirmed the overall benefit of multimodal programs but emphasized the difficulty in isolating the contribution of individual components.

The timing of the intervention remains a key point of discussion. Gillis et al. (13) demonstrated that prehabilitation may be more effective than postoperative rehabilitation in improving functional outcomes. Our results support this perspective: by initiating the intervention at baseline (T−4), we observed benefits that persisted up to 12 months (T + 52). Notably, patients in the intervention group started with higher baseline anxiety levels yet achieved lower long-term scores compared with controls, suggesting that prehabilitation may be particularly valuable for patients presenting with greater initial psychological vulnerability.

Despite these positive findings, several challenges remain in translating prehabilitation into routine clinical practice. Heil et al. (34) identified organizational barriers and issues related to patient adherence as key limitations. Embedding prehabilitation within an existing ERAS pathway may help address some of these issues by creating a more integrated and continuous model of care. Although the reduction in the Comprehensive Complication Index (CCI) in our intervention group did not reach statistical significance, the observed trend may reflect improved overall resilience and recovery.

Some limitations should be acknowledged. Baseline differences in anxiety and functional status between groups may introduce a degree of selection bias, although the divergent trajectories observed over time suggest a relevant effect of the intervention. In addition, the exclusion of patients with clinically significant anxiety or depression limits the applicability of these findings to more vulnerable populations. Finally, the relatively small sample size and the single-center design indicate the need for larger, multicenter randomized studies, in line with CONSORT recommendations, to confirm and extend these results. A relevant limitation of the present study is the preliminary and exploratory nature of the analysis. No formal sample size calculation was performed specifically for the psychological and quality-of-life endpoints. The relatively small sample size limits statistical power, particularly for secondary outcomes and subgroup analyses. The absence of a formal sample size calculation specifically powered for psychological and HRQoL endpoints means that non-significant findings, especially for depressive symptoms, should be interpreted cautiously. Accordingly, the present findings should be regarded as preliminary and hypothesis-generating. In addition, the statistical approach was primarily based on longitudinal within-group analyses and exploratory between-group comparisons at individual time points, without the application of multivariable longitudinal models or linear mixed models. Therefore, the results should be interpreted with caution and require confirmation in adequately powered multicentre studies. Accordingly, the present findings should be regarded as preliminary and hypothesis-generating.

## Conclusion

Trimodal prehabilitation integrated within an ERAS pathway may be associated with more favourable anxiety trajectories and improved perceived recovery in patients undergoing colorectal cancer surgery. These findings underscore the potential importance of systematically addressing psychological and functional domains within contemporary perioperative care models. However, given the exploratory nature of the analysis and important methodological limitations, including the small sample size and the absence of formal longitudinal interaction modelling, these results should be interpreted with caution and considered hypothesis-generating.

Future studies should focus on adequately powered, multicentre randomized controlled trials employing robust longitudinal statistical approaches to validate these preliminary findings and to better delineate the role of trimodal prehabilitation within oncologic surgical pathways. Furthermore, there is a need to investigate more personalized and adaptive intervention strategies, potentially supported by digital health tools, as well as to assess the durability of the observed benefits over extended follow-up periods.

## Data Availability

The raw data supporting the conclusions of this article will be made available by the authors, without undue reservation.
